# Morphology, Photosynthetic Traits, and Nutritional Quality of Lettuce Plants as Affected by Green Light Substituting Proportion of Blue and Red Light

**DOI:** 10.3389/fpls.2021.627311

**Published:** 2021-07-07

**Authors:** Lie Li, Yu-xin Tong, Jun-ling Lu, Yang-mei Li, Xin Liu, Rui-feng Cheng

**Affiliations:** ^1^Institute of Environment and Sustainable Development in Agriculture, Chinese Academy of Agricultural Sciences, Beijing, China; ^2^Key Laboratory of Energy Conservation and Waste Management of Agricultural Structures, Ministry of Agriculture, Beijing, China

**Keywords:** lettuce (*Lactuca sativa* cv. ‘Tiberius’), green light, shade avoidance syndrome, light transmittance, stomata characteristics, plant photosynthesis, plant morphology

## Abstract

Green light, as part of the photosynthetically active radiation, has been proven to have high photosynthetic efficiency once absorbed by plant leaves and can regulate plant physiological activities. However, few studies have investigated the appropriate and efficient way of using the green light for plant production. Thus, the objective of this study was to investigate a moderate amount of green light, partially replacing red and blue light, for plant growth and development. In this experiment, four treatments were set up by adjusting the relative amount of green light as 0 (RB), 30 (G30), 60 (G60), and 90 (G90) μmol m^−2^ s^−1^, respectively, with a total photosynthetic photon flux density of 200 μmol m^−2^ s^−1^ and a fixed red-to-blue ratio of 4:1. Lettuce (*Lactuca sativa* cv. ‘Tiberius’) plant growth and morphology, stomatal characteristics, light absorptance and transmittance, photosynthetic characteristics, and nutritional quality were investigated. The results showed that: (1) shoot dry weight increased by 16.3 and 24.5% and leaf area increased by 11.9 and 16.2% under G30 and G60, respectively, compared with those under RB. Plant stem length increased linearly with increasing green-to-blue light ratio; (2) light transmittance of lettuce leaf under treatments employing green light was higher than that under RB, especially in the green region; (3) stomatal density increased, whereas stomatal aperture area decreased with the increase in the relative amount of green light; and (4) carbohydrate accumulation increased under G60 and G90. Soluble sugar contents under G60 and G90 increased by 39.4 and 19.4%, respectively. Nitrate contents under G30, G60, and G90 decreased by 26.2, 40.3, and 43.4%, respectively. The above results indicated that 15–30% green light replacing red and blue light effectively increased the yield and nutritional quality of lettuce plants.

## Introduction

Plants perceive not only light intensity and photoperiod but also light quality, including monochromatic and polychromatic light, as ambient growth environment signals that induce a large number of physiological responses (Kami et al., 2010). Different light qualities have distinctly different biological effects on plants (Li et al., 2020). There is a misconception that green light is less useful for plant photosynthesis, probably because the light absorption of photosynthetic pigments is relatively low within the green region compared with that within the red and blue regions, especially in the plant canopy (McCree, 1972; Smith et al., 2017). This is also the reason that red and blue light, rather than other lights, are widely used in recently developed plant factories with artificial lighting for plant production (Wang et al., 2016). Moreover, the photoreceptors of red and far-red, blue, and UV-B have been identified as phytochromes, cryptochromes and phototropins, and UV Resistance Locus 8 (Bantis et al., 2018), respectively, but no specific green light photoreceptor was detected in previous studies. Even so, the vital role of green light affecting plant physiological activities was gradually proved in previous studies (Johkan et al., 2012; Wang and Folta, 2013; Materová et al., 2017).

A recently published review suggested that green light distributed energy among plant leaves and canopies (Smith et al., 2017) because green light was capable of reaching deeper and drives CO_2_ fixation under plant canopy, whereas most of the red and blue light is generally absorbed by the upper part of plant leaves (Sun et al., 1998; Schenkels et al., 2020). In addition, a green light could easily induce a shade avoidance syndrome in plants because, in natural conditions, green light transmitted through the plant canopy, causing a sharp decline in blue-to-green (B/G) ratio from the top to the bottom layers of plants (Zadoks et al., 1974). Similar to the shading response caused by far-red light, plant stem elongation and leaf area expansion also occurred under green light (Sellaro et al., 2010; Zhang et al., 2011). Besides, green light shifted cryptochromes from semi-reduced active state caused by blue light to the fully reduced and inactive state. Thus, green light could inactivate blue-mediated responses (Bouly et al., 2007; Liu et al., 2010; Sellaro et al., 2010).

Recent studies suggested that green light should not be ignored in plant growth and development. Kaiser et al. (2019) reported that, by replacing red and blue with green light, the fresh and dry weights of tomato increased linearly with an increase in the percentage of green light. Schenkels et al. (2020) have shown that additional green light significantly increased the total fresh and dry weights of plant seedlings, whereas when replacing red and blue with green light, no significant increase was found in dry weight. The above inconsistent results were mainly caused by the different proportions of green light in the light source. According to McCree (1972), once absorbed by plant leaves, green light showed a higher relative quantum efficiency than blue light. Moreover, the shade avoidance syndrome induced by green light was capable of increasing the stem length and leaf area of plants (Johkan et al., 2012). These changes in plant morphology effectively enhance the photons captured by plant leaves, improving the photosynthetic efficiency (Park and Runkle, 2017). Thus, investigating a moderate proportion of green light can optimize the photosynthesis of plant leaves to maximize the yield in plant production.

Therefore, this study aimed to investigate the optimal proportion of green light for lettuce growth and development by adjusting the relative amount of green light. Combined red and blue light were used as the fundamental light. Lettuce (*Lactuca sativa* cv. ‘Tiberius’) was used in this experiment because it is one of the most popular horticulture vegetables produced in plant factories with artificial lighting. Lettuce morphology, biomass, stomatal characteristics, light absorptance and transmittance, photosynthetic traits, and nutritional quality were evaluated.

## Materials and Methods

### Plant Material and Growth Condition

Lettuce (*Lactuca sativa* cv. ‘Tiberius’) seeds were sown in sponge blocks filled in plastic trays. Seedlings were developed under fluorescent lamps (TL-D56W, Osram, Munich, Germany) with a light intensity of 150 μmol m^−2^ s^−1^ and a photoperiod of 16 h day^−1^. Upon emerging three true leaves (15 days after sowing), seedlings were transplanted in a plant factory with artificial lighting (PFAL), in which the day/night temperature was 24/20°C, relative humidity was 60–70%, and the concentration of CO_2_ (supplied by a CO_2_ gas cylinder) was 1,000 μmol mol^−1^. Plants were cultivated in a customized circulation system of nutrient solution with a planting density of 32 plants m^−2^. The light-emitting diode (LED) panels (Datang New Energy Technology Co., Ltd., Shenzhen, China) were equipped on cultivation shelves. The distance between the LED panels and the culture bed was 0.3 m. An opaque white plastic reflective film was placed around the LED panels to ensure uniform radiation on the surface of the culture beds and to prevent light pollution from the adjacent treatments. Modified Yamasaki nutrient solution (EC = 1.2 dS m^−1^, pH = 5.8) was applied for the plant growth. The circulation system was automatically operated for 1 h day^−1^.

### Experimental Setup

**Figure 1:**
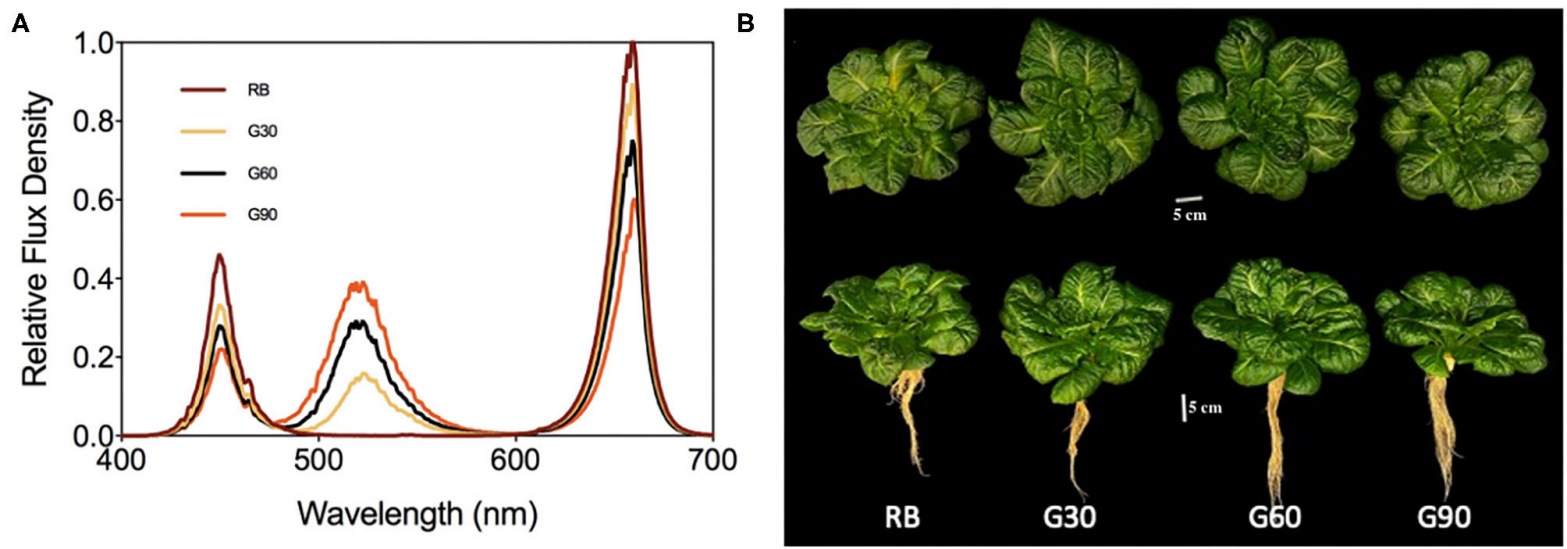
The spectral distribution of different treatments **(A)** and photograph of lettuce grown under treatments **(B)**.

The photosynthetic photon flux density (PPFD) and red (600–690 nm, peak at 660 nm) to blue (410–490 nm, peak at 450 nm) (R/B) ratio of all treatments were maintained at 200 μmol m^−2^ s^−1^ and 4:1, respectively. The relative amount of green light (490–590 nm, peak at 525 nm) of each treatment was adjusted to 0 (RB), 30 (G30), 60 (G60), and 90 (G90) μmol m^−2^ s^−1^ (Table 1). The light spectrum of each treatment was presented in Figure 1. Plants were harvested on day 20 after transplanting. This experiment was repeated three times.

**Table 1:** Different light intensity (μmol m^−2^ s^−1^) of red, blue, and green light of four treatments.

**Treatments**	**Red**	**Blue**	**Green**	**PPFD^a^**	**YPFD^b^**
RB	160	40	0	200	178.8
G30	136	34	30	200	173.9
G60	112	28	60	200	168.9
G90	88	22	90	200	164.0

a *PPFD, Photosynthetic photon flux density, μmol m^−2^ s^−1^*.

b *YPFD, Yield photon flux density, which is the product of spectral photon distribution and relative quantum efficiency according to Sager et al. (1988) and McCree (1972)*.

### Determinations of Plant Growth and Morphology

For the growth and morphology analysis, destructive measurements were taken on five plants of each treatment on day 20 after transplanting. Lettuce samples were separated into shoot and root using sharp scalpels and forceps. The stem length was the average value of stem lengths in fully expanded leaves of each plant. The leaf area was measured by an area meter (LI-3100C, LI-COR Biosciences, Lincoln, NE, USA). The fresh and dry weights of shoot and roots were measured by an electronic balance (Si-234; Denver Instrument, Bohemia, NY, USA). Whole-plant net assimilation was calculated by dividing shoot dry weight by total leaf area for each plant.

### Measurements of Light Absorptance and Light Transmittance of Leaves

Light reflectance (Rf) and light transmittance (Tr) were measured on the fully expanded second leaves with a spectroradiometer (Ocean Optics USB2000+, Dunedin, FL, USA) in combination with two integrating spheres (FOIS-1, ISP-REF, Ocean Optics Inc., Dunedin, FL, USA). Light absorptance (Ab) was calculated as: Ab = 1 – (Rf + Tr). The diagram of the measuring apparatus is showed in Supplementary Figure 1.

### Observation of Stomata

Leaf samples were collected from the fully expanded second young leaves of five plants in the same region of each treatment. Leaf stomatal characteristics were measured using the method of Wang et al. (2016). The stomata of plants are regarded as rhombus. Stomatal aperture area was calculated by long axis length of aperture (Al) and short axis length of aperture (As). Stomatal aperture area = Al × As/2.

### Determinations of Photosynthetic Pigments and Photosynthetic Characteristics

From the top to the bottom, the leaves of 2, 4, 6, 8, 10, 12, 14, 16, 18, and 20 from four plants in each treatment were collected at the same point with 1 cm^2^ by using a punch. Subsequently, 96% ethanol was used as a solvent for these samples to measure chlorophyll a (Chl a), chlorophyll b (Chl b), and carotenoid content. The absorbance was measured at 665, 649, and 470 nm by using a spectrophotometer (UV-1800, Shimadzu Corp., Kyoto, Japan). Concentrations of chlorophyll and carotenoid were determined using the equations reported by Lichtenthaler and Wellburn (1983). All of the data measured were used for the normal distribution analysis shown in Figure 7.

Net photosynthetic rate (P_n_), gas exchange (g_s_), and intercellular CO_2_ concentration (Ci) of lettuce fully expanded second leaves were measured using a portable photosynthetic instrument (LI-6400XT, LI-COR Biosciences, Lincoln, NE, USA) with a transparent leaf chamber (6400-08, LI-COR Biosciences, Lincoln, NE, USA) under the actual light condition of plant growth. The environmental conditions of the leaf chamber were maintained at 24°C, 1,000 μmol m^−2^ s^−1^ CO_2_ level, and 60–70% relative humidity (Li et al., 2020). The order of measurements was arranged randomly for each repetition, and each treatment was repeated three times. P_n_ and g_s_ of lettuce leaves measured in LED leaf chamber were represented by P_n−T_ and g_s−T_, respectively.

P_n_ and g_s_ to light intensity response curves were taken on the fully expanded second leaves by using the same portable photosynthetic instrument (LI-6400XT, LI-COR Biosciences, Lincoln, NE, USA) with a red–blue LED leaf chamber (6400-02B, LI-COR Biosciences, Lincoln, NE, USA) installed. The leaf chamber temperature, the concentration of CO_2_, relative humidity, airflow rate, and leaf-to-air vapor pressure difference (VPD_leaf−air_) in the leaf chamber were 24°C, 1,000 μmol mol^−1^, 60–70%, 500 μmol s^−1^, and 1.0 ± 0.1 kPa, respectively. P_n_ and g_s_ under different light intensities were measured subsequently. The starting light intensity was 200 μmol m^−2^ s^−1^, followed by 100, 0, 200, 400, 600, 800, 1,000, and 1,200 μmol m^−2^ s^−1^. Light intensity decreasing from the actual growth level to dark and then gradually increasing to high light intensities was needed when measuring light intensity-photosynthesis response curves of plant leaves (Wang et al., 2016). The light source was red and blue light with an R/B ratio of 4:1. P_n_ and g_s_ were recorded when P_n_ reached a steady state at each light intensity. Measurement order was arranged randomly for every repetition, and each treatment was repeated three times. P_n_ and g_s_ of lettuce leaves measured in LED leaf chamber were represented by P_n−L_ and g_s−L_, respectively.

### Determinations of Sucrose, Starch, and Nutritional Quality

Lettuce leaves were collected in sample bottles, quick-frozen by using liquid nitrogen, and stored in an ultralow temperature freezer before the end of the dark period. Five plants of each treatment were taken at the end of the light period on day 20 after transplanting. The samples were used to measure sucrose, starch, and nutritional quality contents, including soluble sugar, nitrate, and crude fiber. Sucrose content was determined using the method described by Fils-Lycaon et al. (2011) and measured at 480 nm. Starch content was measured and calculated according to Clegg (1956). Soluble sugar content was determined using the method described by Fairbairn (1953). Nitrate content was determined by the method described by Cataldo et al. (1975). The ELISA performed by using the double-antibody one-step sandwich method was used to determine the crude fiber content of lettuce leaves. The absorbance was measured at 450 nm wavelength with a spectrophotometer (Shimadzu UV-1800, Shimadzu Corp., Kyoto, Japan).

### Statistical Analysis

This experiment was designed as a single factor experiment. A one-way ANOVA was performed for each treatment. When the ANOVA result was significant, Duncan's multiple range test at *p* < 0.05 was used for mean separation. The linear relationships between plant stem length and G/B ratio were determined by GraphPad Prism 8 (GraphPad Software Inc., San Diego, CA, USA), using simple linear regression. The fitting light intensity response curves of P_n_ and g_s_ with non-linear regression were performed by the GraphPad Prism 8 (GraphPad Software Inc., San Diego, CA, USA), using [Agonist] vs. response–variable slope (four parameters) equation. The data of chlorophyll concentration in different layers of lettuce plants were performed by a box-plot and drawn with the Origin 2020 software (OriginLab Inc., Northampton, MA, USA). The desperation of data is proportional to the height of the box. The lines and dots in the box represent the median and the average values, respectively. The highest and the lowest point of the vertical line represent the maximum and minimum values. All statistical analyses were performed by the IBM SPSS Statistics 26 program (SPSS Inc., Chicago, IL, USA). This experiment was repeated three times.

## Results

### Growth and Morphology

**Figure 2:**
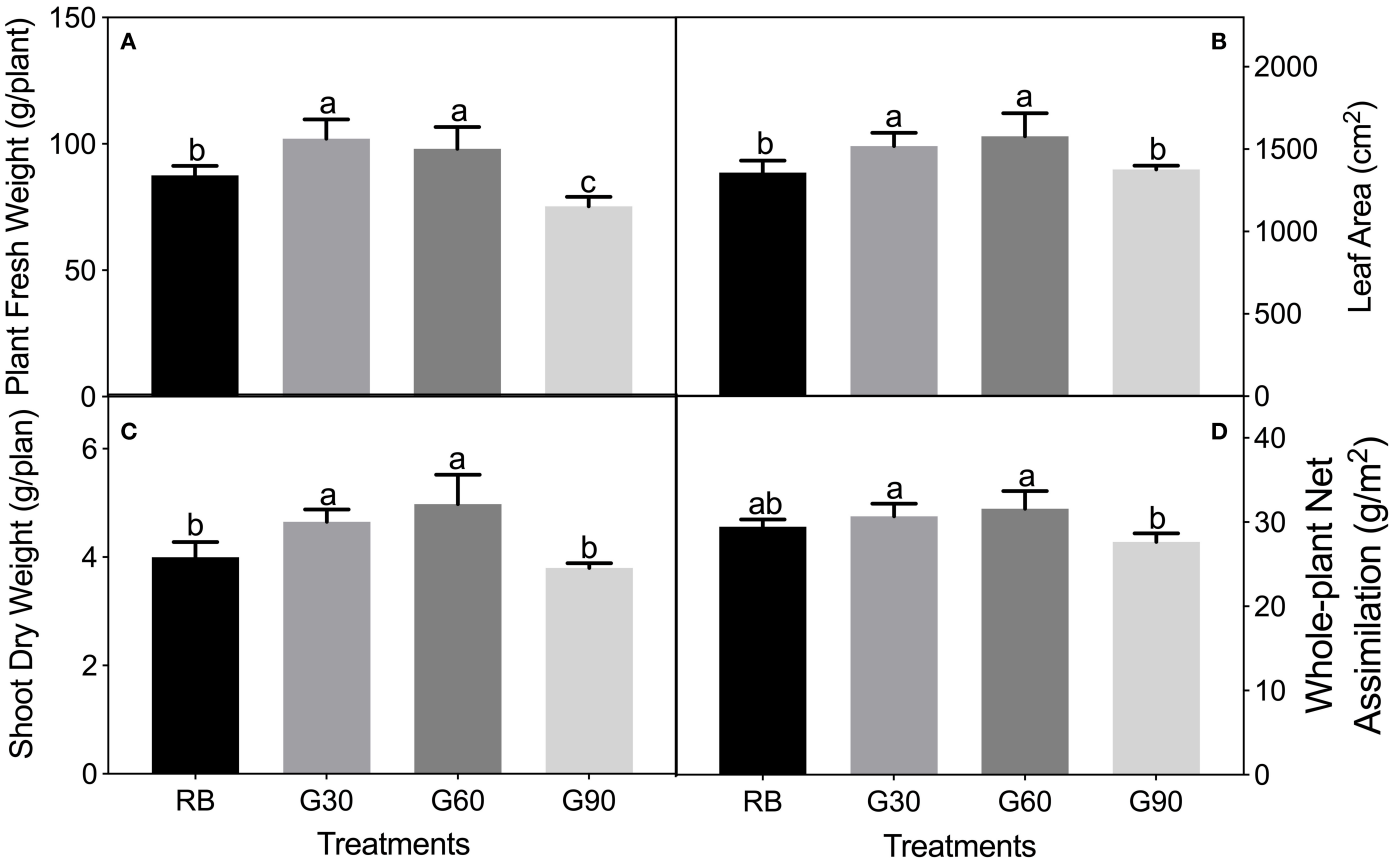
Plant fresh weight **(A)**, leaf area **(B)**, shoot dry weight **(C)**, and whole-plant net assimilation **(D)** of lettuce plants as affected by the different proportions of green light. Different letters in each column indicate significant differences at *p* < 0.05, according to the least significant difference (LSD) test.

**Figure 3:**
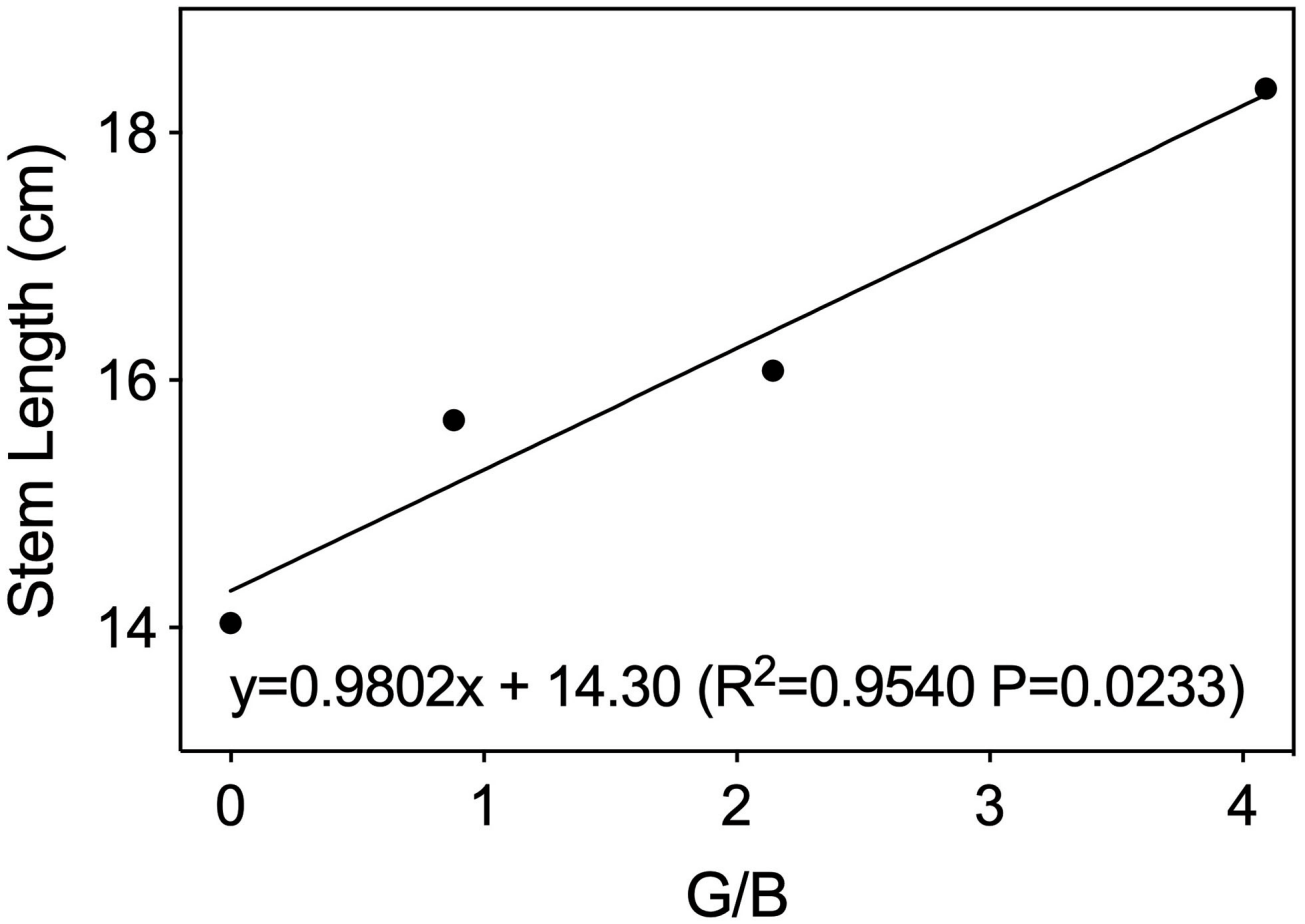
Stem length of lettuce leaves as affected by green-to-blue (G/B) ratio. Associated correlation of coefficients (*R*^2^) and regression equations presented here indicate significant differences at *p* < 0.05.

As shown in Figure 2, plant fresh weight, leaf area, and shoot dry weight were 12–25% greater under G30 and G60 than those under RB or G90. The increased leaf area accounted for most of the increased plant mass so that the trend of whole-plant net assimilation was nearly constant with the leaf area among four treatments. The stem length of lettuce increased linearly with increasing G/B ratio (Figure 3).

### Light Absorptance and Light Transmittance of Lettuce Leaves

**Figure 4:**
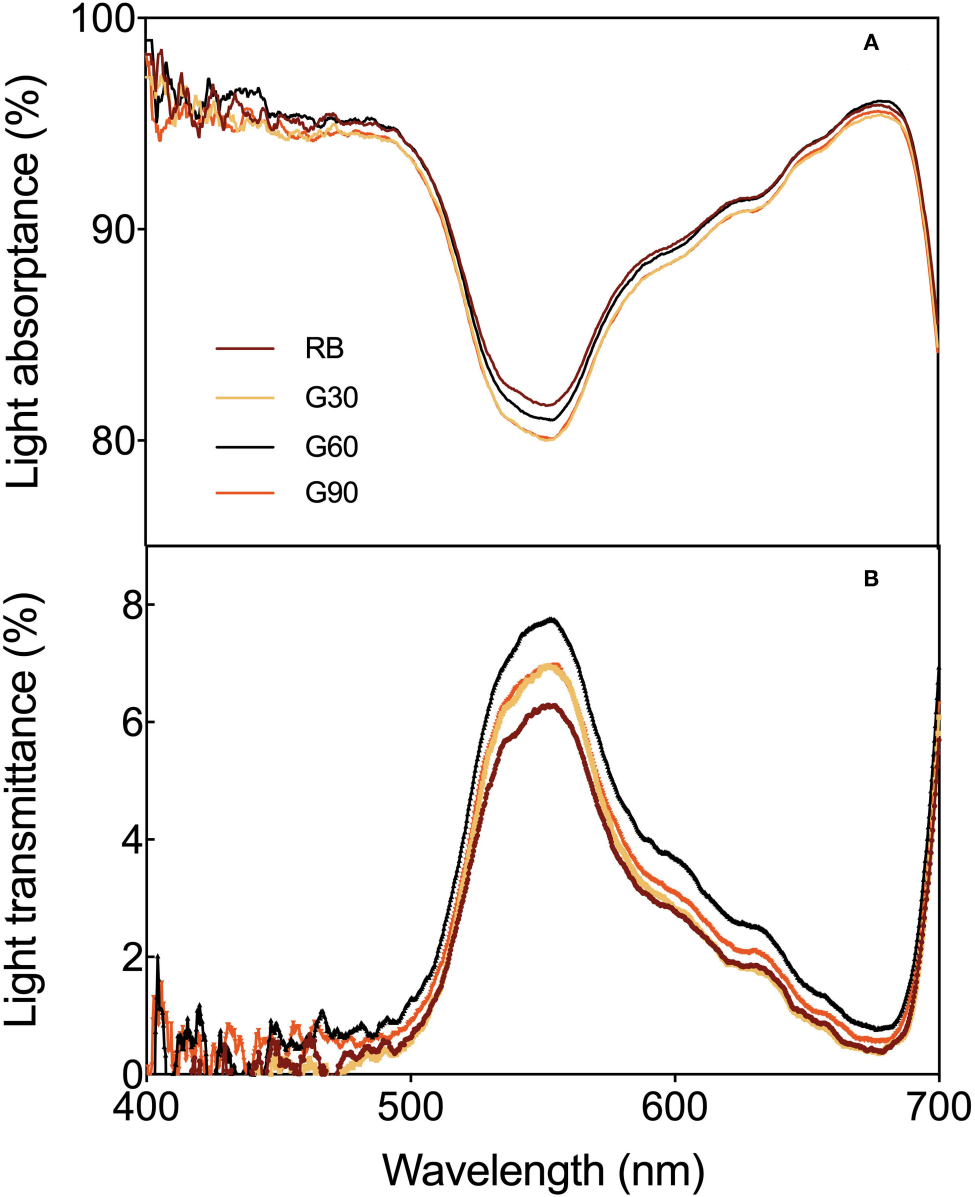
Light absorptance **(A)** and light transmittance **(B)** as affected by the different proportions of green light.

As shown in Figure 4, the lowest light absorptance and highest light transmittance of lettuce leaves were observed in the green range within the visible spectrum of 400–700 nm. Meanwhile, the light transmittance was higher under treatments employing green light than that under RB, whereas the light absorptance was, on the contrary, especially within the green region. The minimal absorptance and maximal transmittance found at the same wavelength point (555 nm) were 80% under G30 and 8% under G60, respectively.

### Stomatal Characteristics

**Figure 5:**
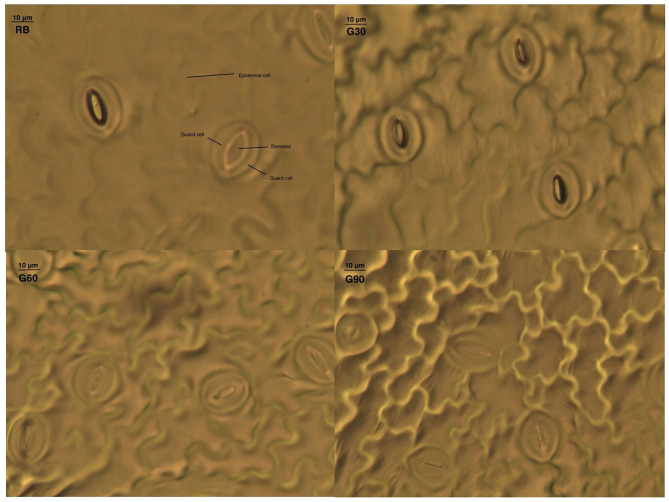
Photos of stomatal characteristics under the different proportions of green light.

As shown in Table 2, the stomatal development was significantly affected by the relative amount of green light. Stomatal density increased by 47, 38, and 71% under G30, G60, and G90, respectively, compared with that under RB, whereas epidermal cell density only increased under G90. With the increase in green proportion from 15 to 45%, the long axis length and short axis length of aperture decreased by 7–25% and 14–51%, respectively. The reduction of long and short axis length of aperture caused a linear decrease in aperture area from 45.1 to 16.5 μm^2^ with an increase in the relative amount of green light (Figure 5).

**Table 2:** Effects of green light on stomatal characteristics of lettuce.

	**RB**	**G30**	**G60**	**G90**
Stomatal density (N mm^−2^)	117.2 ± 26.2^c^	172.5 ± 25.0^b^	161.7 ± 29.4^b^	200.0 ± 21.9^a^
Epidermal cell density (N mm^−2^)	717.2 ± 158.0^b^	662.5 ± 75.9^b^	700.8 ± 99.6^b^	1007.0 ± 89.1^a^
Long axis length of aperture (μm)	16.2 ± 2.1^a^	15.0 ± 2.0^b^	14.3 ± 2.1^b^	12.2 ± 2.6^c^
Short axis length of aperture (μm)	5.6 ± 1.2^a^	4.8 ± 1.3^b^	3.8 ± 1.2^c^	2.7 ± 1.7^d^
Aperture area (μm^2^)	45.1 ± 1.3^a^	35.9 ± 1.4^b^	26.9 ± 1.3^c^	16.5 ± 2.0^d^

*The data are means ± SDs (n = 5). Different lowercase letters in the same line show significant difference at p < 0.05, according to the least significant difference (LSD) test*.

### Leaf Photosynthetic Pigments

**Figure 6:**
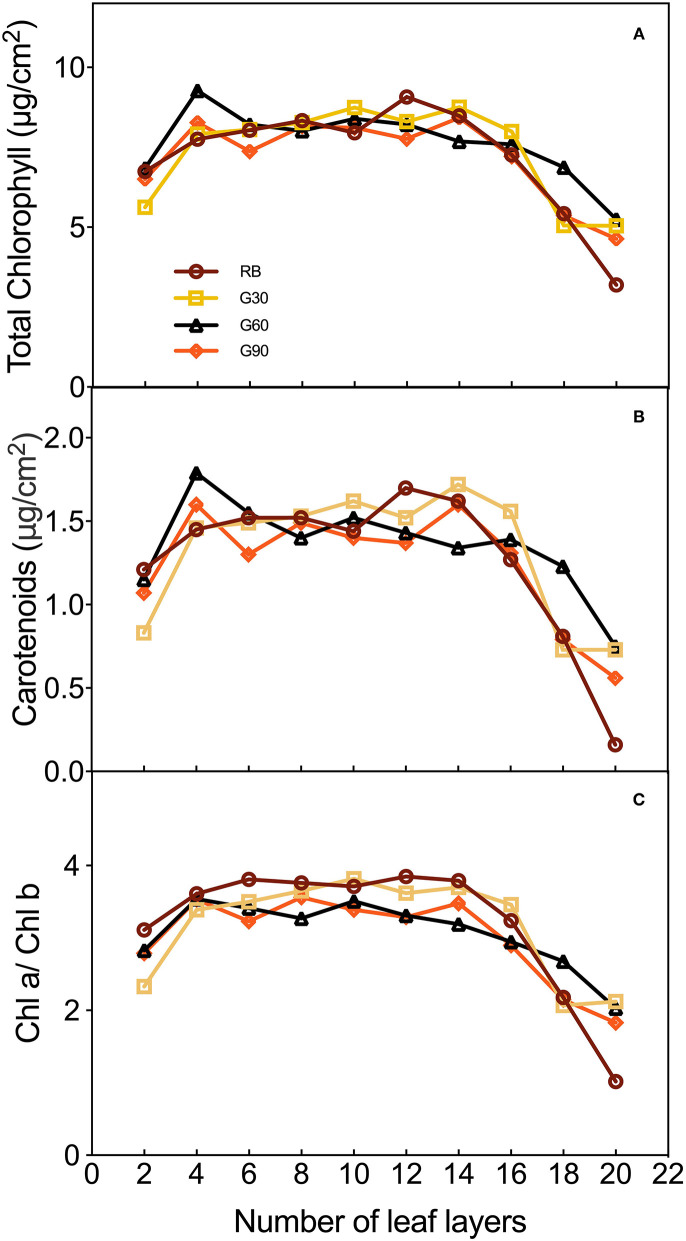
The concentrations of total chlorophyll **(A)**, carotenoids **(B)**, and of leaves and Chl a/Chl b **(C)** of different layers of lettuce plants as affected by the different proportions of green light.

The concentrations of total chlorophyll and carotenoids, and Chl a/Chl b in different leaf layers of lettuce were different. As shown in Figure 6, these three pigment parameters increased dramatically from the 2nd to the 4th leaf. Meanwhile, the total chlorophyll and carotenoids under G60 and G90 reached their peak at the 4th leaf, whereas they decreased dramatically at the 6th leaf. Then, the curves of the three pigment parameters were relatively flat under G30, G60, and G90 from the 6th to the 14th leaf, and gradually dropped from the 14th to the 20th leaf. A significantly decrease under RB was observed on the three pigment parameters from the 12th to the 20th leaf and finally reached the lowest level among all treatments.

**Figure 7:**
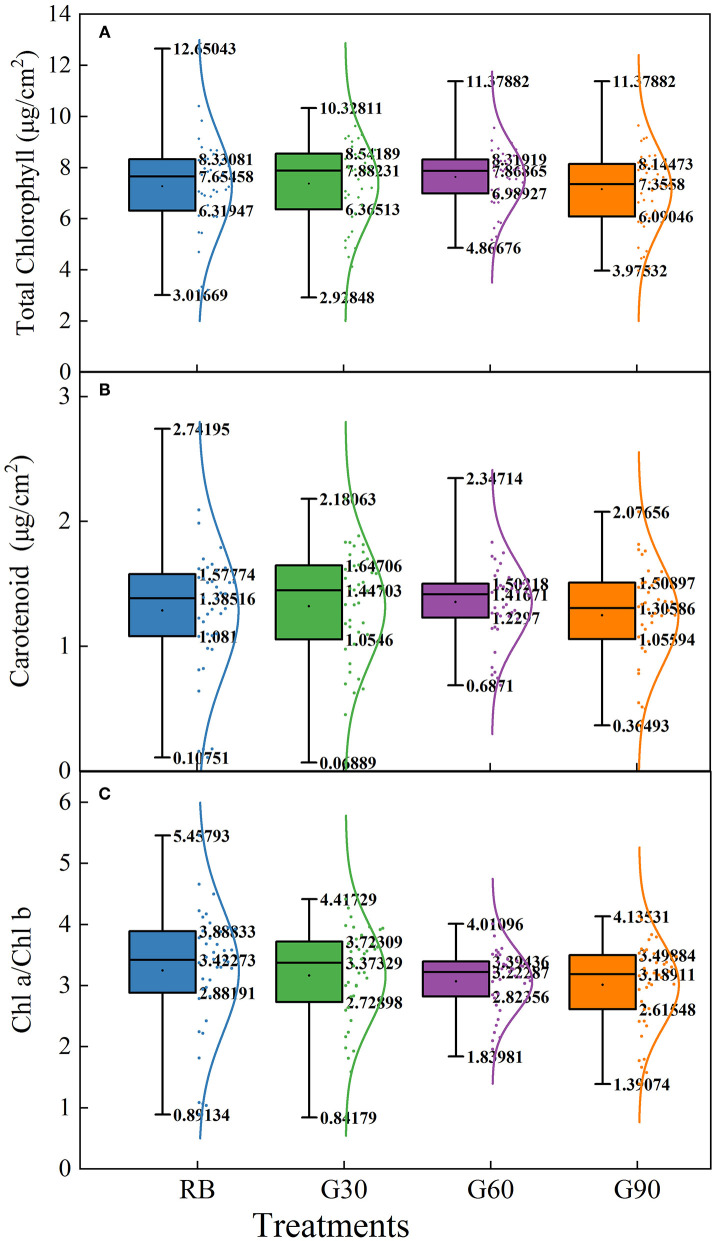
Box-plot and normal distribution of concentrations of total chlorophyll **(A)**, carotenoid **(B)**, and Chl a/Chl b **(C)** of different layers of lettuce under different proportions of green light.

As shown in Figure 7, the data of the three pigment parameters of lettuce leaves in different layers under G60 are the most compact in all treatments, whether from the difference between the maximum and minimum values or the height of the box. Employing green light, the difference between maximum and minimum values was decreased significantly, though the highest values of the three pigment parameters were observed under RB. These results suggested that green light reduced the gap of the concentrations of pigments between upper and lower leaves of plants, especially when green light accounts for 30% (G60) in the light source.

### Leaf Photosynthetic Traits

**Figure 8:**
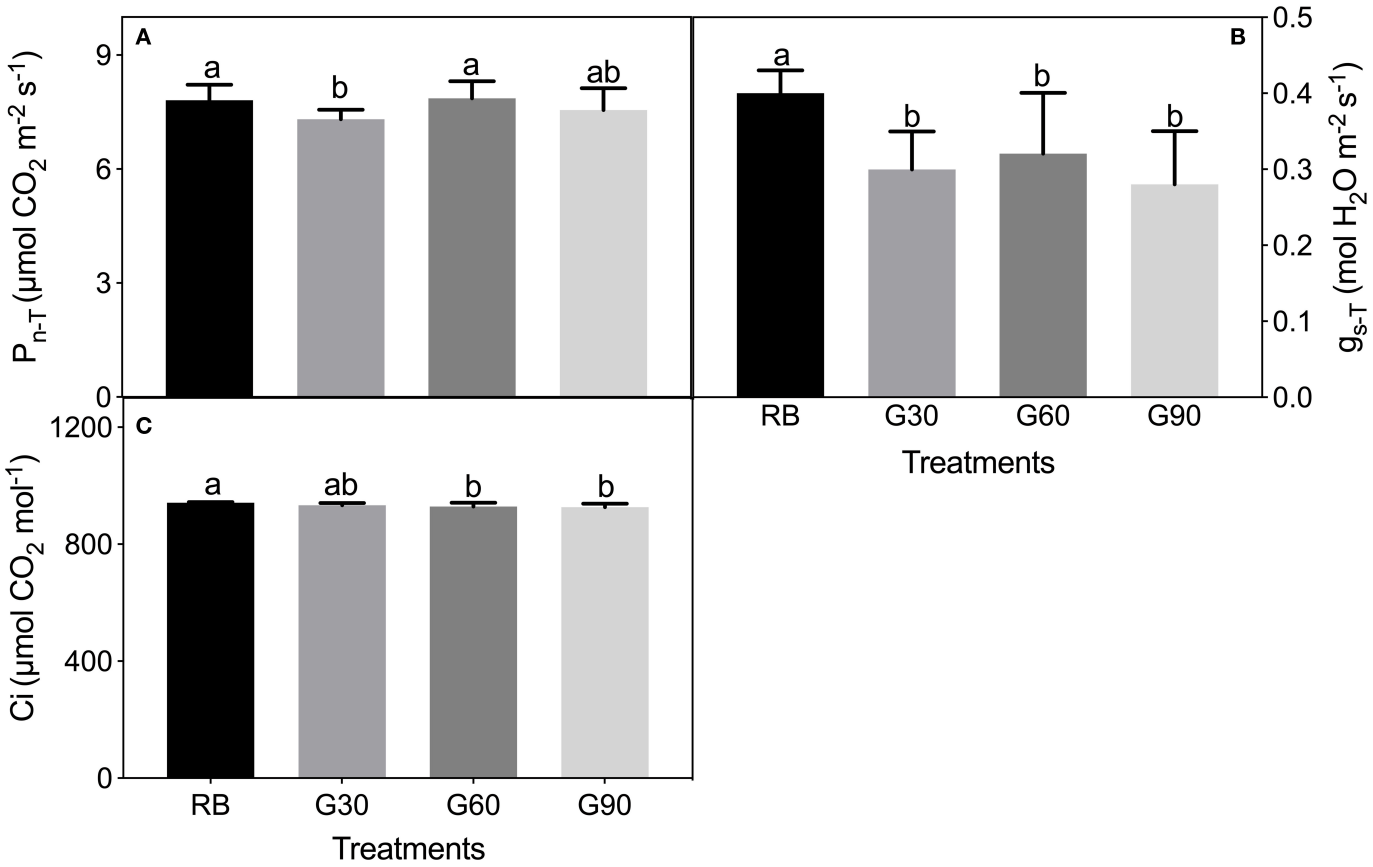
P_n−T_ **(A)**, g_s−T_ **(B)**, and Ci **(C)** under the actual light condition of lettuce leaves as affected by different proportions of green light. Different letters in each column indicate significant differences at *p* < 0.05, according to the least significant difference (LSD) test.

P_n−T_ under G30 decreased by 6% compared with that under RB, whereas no significant difference in P_n−T_ was found among RB, G60, and G90. g_s−T_ and Ci under treatments employing green light decreased significantly compared with those under RB (Figure 8).

**Figure 9:**
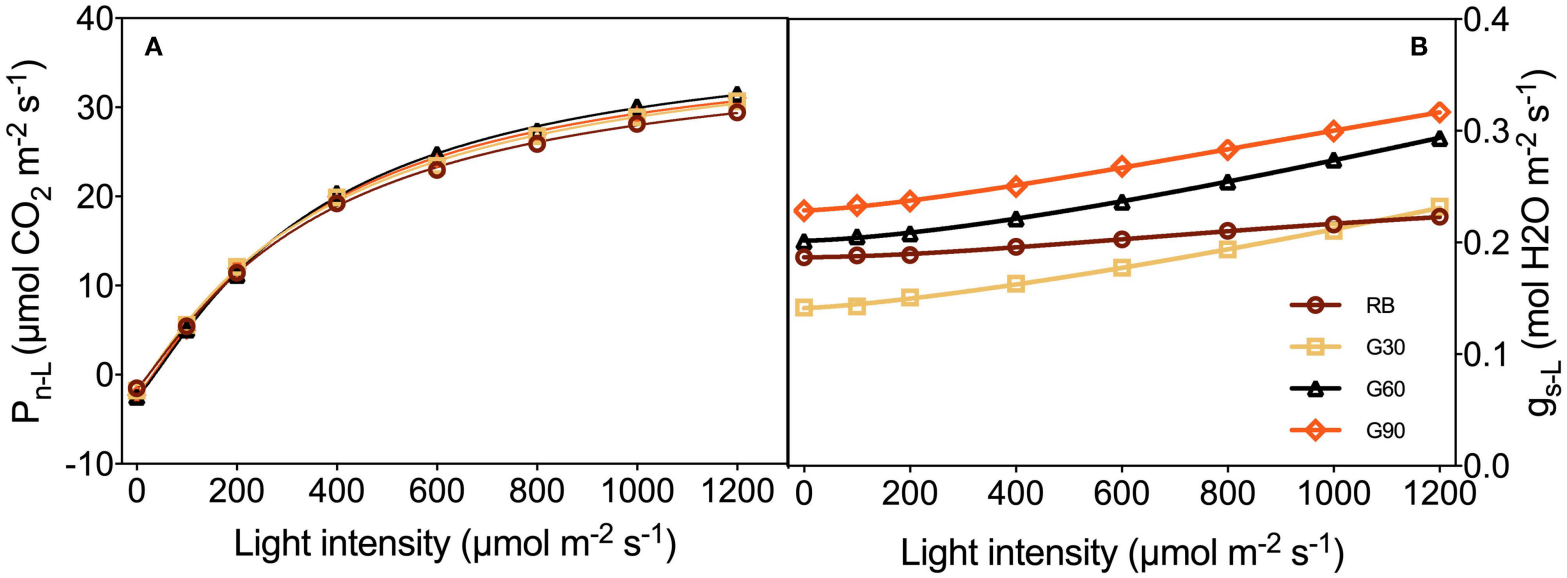
P_n−L_ **(A)** and g_s−L_ **(B)** to light intensity response curves as affected by the different proportions of green light.

As shown in Figure 9, P_n−L_ and g_s−L_ increased with the increasing light intensity. Among treatments employing green light, g_s−L_ increased with an increase in the relative amount of green light. Under G30, g_s−L_ was lower than that under RB, especially when light intensity was lower than 1,000 μmol m^−2^ s^−1^, whereas g_s−L_ under G60 and G90 was higher than that under RB. There was no obvious difference in P_n−L_ among all treatments under the same light intensity (Figure 9).

### Sucrose, Starch, and Nutritional Quality

As shown in Table 3, an increase in the carbohydrate accumulation of lettuce leaves was found under treatments employing green light compared with that under RB. Sucrose and starch contents increased by 60 and 37% under G60 and by 35 and 40% under G90, respectively, compared with those under RB. In terms of nutritional quality, soluble sugar contents under G60 and G90 increased by 39 and 19%, respectively, compared with that under RB. Nitrate contents of lettuce leaves significantly decreased under treatments employing green light. Nitrate contents under G30, G60, and G90 decreased by 26, 40, and 43%, respectively, compared with that under RB. No significant differences in crude fiber contents were found among all treatments.

**Table 3:** The accumulation of carbohydrate and nutritional quality as affected by different relative amount of green light.

	**RB**	**G30**	**G60**	**G90**
Sucrose (mg g^−1^)	4.3 ± 1.1^b^	5.4 ± 1.5^ab^	6.9 ± 1.7^a^	5.8 ± 1.7^ab^
Starch (mg g^−1^)	1.8 ± 0.2^b^	2.0 ± 0.5^b^	2.8 ± 0.7^a^	2.9 ± 0.7^a^
Soluble sugar (mg g^−1^)	16.1 ± 1.8^b^	15.4 ± 1.7^b^	22.5 ± 1.7^a^	19.24 ± 6.6^ab^
Nitrate (mg g^−1^)	1.8 ± 0.3^a^	1.4 ± 0.2^b^	1.2 ± 0.3^c^	1.2 ± 0.4^c^
Crude fiber (mg g^−1^)	8.9 ± 2.9^a^	10.3 ± 2.3^a^	9.9 ± 3.4^a^	9.9 ±2.5^a^

*The data are means ± SDs (n = 5). Different lowercase letters in the same line show significant difference at p < 0.05, according to the least significant difference (LSD) test*.

## Discussion

### Morphology of Lettuce Plants as Affected by Employing Green Light

Light quality, as energy and signal sources, significantly affects plant photomorphogenesis (Bantis et al., 2018). Similar to red/far-red (R/FR) ratio, the G/B ratio also could act as a shade signal within a plant canopy and could provide information about the degree of shading, triggering physiological and morphological changes of plants to best intercept available energy (Sellaro et al., 2010; Smith et al., 2017). In this experiment, lettuce stem length increased with increasing G/B ratio. This result was consistent with the finding that the hypocotyl length of Arabidopsis seedlings increased linearly with increasing G/B ratios within the range of 0.5–1.0 while cultivated in a natural environment (Sellaro et al., 2010). The above phenomenon can be explained by the shade avoidance syndrome induced by the green light. As shown in Figure 4, the light transmittance in the green region was higher than that in the blue region so that an increase in G/B ratio was induced under the lettuce canopy, providing a shade signal for plants. The shade signal can be detected by relevant non-photosynthetic photoreceptors, and it can then induce the shade avoidance syndrome, such as stem elongation and leaf expansion, to intercept more available light energy (Wang and Folta, 2013). Kang et al. (2016) also found that green light increased the leaf length in lettuce plants. Moreover, leaf expansion was also found under G30 and G60 in this study. This was consistent with the finding that lettuce leaf area increased dramatically when green light replaced red and blue light (Kim et al., 2004). This kind of morphological changes caused by a high G/B ratio may be attributed to cryptochromes or an unknown role of phytochromes, or perhaps a novel light photoreceptor (Sellaro et al., 2010; Zhang et al., 2011). However, the leaf area under G90 showed no significant difference with RB. This may be caused by the low yield photon flux density (YPFD) in G90 (Johkan et al., 2012).

### Gas Exchange of Lettuce Leaves as Affected by Employing Green Light

Stomatal conductance was mainly affected by stomatal density and the degree of stomatal opening (Fanourakis et al., 2015). Previous studies have shown that stomatal density in newly developed leaves of C3 plants is independent of their adjacent irradiance (Lake et al., 2001; Miyazawa et al., 2006). On the contrary, exposure of mature leaves to low light conditions triggered long-distance signaling that controls the stomatal development and caused a reduction in stomatal density in young leaves (Ehonen et al., 2020). This phenomenon is called systemic signaling (Lake et al., 2001). In this experiment, stomatal density increased with an increase in the relative amount of green light. This should be attributed to green light showing a higher light transmittance to plant canopy in comparison with red and blue light. Meanwhile, the local light intensity in mature leaves of relative lower layers increased with the increasing relative amount of green light. The above result was consistent with the finding that stomatal density increased significantly when blue LEDs were replaced with green LEDs in green leaf lettuce (Son and Oh, 2015).

Green light can reverse the stomatal openings stimulated by blue light (Frechilla et al., 2000), because the fully oxidized state of the flavin chromophore of cryptochrome in darkness is shifted to a semi-reduced form by blue light, and this semi-reduced form is converted to a reduced form particularly by absorbing green light (Liu et al., 2010). This was the reason why stomatal aperture decreased linearly with increasing G/B ratio in this study. This was also the main reason that caused lower g_s−T_ under treatments employing green light than that under RB. However, the stomatal opening could be restored if the green light was followed or replaced by a second blue light in a response analogous to the R/FR reversibility of phytochrome responses (Frechilla et al., 2000; Taiz and Zeiger, 2015). In this research, the stomatal density of lettuce leaves caused by systemic signaling could not change immediately with the change of light quality; thus, under the same red and blue light condition, g_s−L_ increased with an increase in the relative amount of green light. The above phenomenon gave an efficient way to regulate stomatal behaviors. In future experiments, green light can be considered to increase the stomatal density at the early growth stage of plant leaves, whereas blue light can be considered to increase the stomatal opening at the yield-forming stage of plants so that, especially in plant factories, plant yield can be increased efficiently. Previous studies also demonstrated that stomatal response to the light environment could provide potential targets for increasing photosynthesis, water use efficiency, and overall plant yield (Matthews et al., 2020).

### Green Light Affected Pigmentation, Light Energy Distribution, and Photosynthetic Characteristics in Lettuce

Most of the previous studies mainly focused on the pigment contents of individual leaves rather than the whole plant. In this study, pigment concentrations of the lettuce leaves in different layers were measured to investigate the effect of green light on pigment concentrations of leaves within the canopy. The light absorptance of red and blue light was higher, but the light transmittance was lower in the upper leaves of lettuce. Thus, the relatively scattered pigment concentrations and Chl a/Chl b were found under RB. Compact distribution of pigment concentrations and Chl a/Chl b under G60 was mainly caused by the maximal light transmittance found under G60, which helped the pigmentation of lower layer leaves of lettuce plants in this study. This was beneficial for whole-plant photosynthesis. In comparison with G60, the lower pigment level of bottom leaves under G30 may be due to the relative lower green light intensity, limiting the irradiance to transmitting through the canopy, whereas under G90, they may be related to a stronger shade condition caused by a higher G/B ratio (Smith and Whitelam, 1997). These results suggested that a moderate amount of green light can promote the pigmentation of underlying leaves in the lower layer canopy.

Green light was capable of contributing light energy to photosynthesis both at the leaf level and the canopy level of plants (Smith et al., 2017). Up to 80% of green light was detected to pass through chloroplast, when 15–25% of green light was considered to transmit through the canopy of plants, efficiently promoting green light absorbed by chloroplasts either in the deeper mesophyll of a single leaf or lower leaves within the canopy, especially in plants with a folded structure, such as lettuce (Terashima et al., 2009). Relative lower light absorptance and higher light transmittance in the green region were also found in this experiment, in comparison with red and blue light. Moreover, higher light transmittance was observed in lettuce leaves under treatments employing green light compared with that under RB, especially in green regions. This result suggested that green light promoted more light energy to transmit through leaves. However, no significant increase in the light transmittance was found by using green light in *Ocimum basilicum* L. (Schenkels et al., 2020). This inconsistent phenomenon may be because the green light accounted for only 12% of the light source in this previous study.

Green light can be transmitted into deeper mesophyll of leaves and promote photosynthesis. In addition, once absorbed by chloroplasts, green light could drive photosynthesis with the close quantum efficiency of red and blue light (McCree, 1972; Smith et al., 2017). Thus, though the YPFD, which has been used to quantify the net radiation driving photosynthesis, of G60 and G90 was lower than that of RB, no significant differences of P_n−T_ among RB, G60, and G90 were found in this research. However, P_n−T_ under G30 was found to be lower than that under RB. This may be because the YPFD of G30 was lower than that of RB, and the relatively lower amount of green light was not enough to promote photosynthesis in the deep layer of leaves. The relatively lower light absorption found under G30 was also the cause of the lowest P_n−T_ found under G30 in this study. When transferred to a similar red–blue light source, lettuce leaves of four treatments showed no differences on P_n−L_, though g_s−L_ differed. This was mainly because g_s_ was no longer the limiting factor for plant photosynthesis under high CO_2_ levels.

### Moderate Amount of Green Light Increased the Biomass in Lettuce

Plant dry mass accumulation was mainly determined by the photosynthetic efficiency of plant leaves and total leaf area of plants. The photosynthetic efficiency was primarily affected by the net photosynthetic rate of leaves. However, the net photosynthetic rate (P_n_) under the growth environment did not support the growth results in this study. This phenomenon was also found in previous studies (Kim et al., 2004; Son and Oh, 2015). The possible explanation for this result was that the P_n_ measured on a single leaf cannot represent the P_n_ of the whole plant (Yorio et al., 2001; Li et al., 2020). Thus, whole-plant net assimilation was introduced in this study because whole-plant net assimilation has been used to evaluate the photosynthesis of the whole plant (Park and Runkle, 2018). In this study, the whole-plant net assimilation under G30 and G60 showed a nearly similar trend to the dry mass. This result may indicate that G30 and G60 had the ability to improve the whole-plant photosynthesis of lettuce plants. Moreover, the total leaf area of plants affects the light energy received by the plants, because the incident radiation interception increases with increasing the total leaf area (Bugbee, 2016; Snowden et al., 2016). The promotive effects of G30 (15% green light) and G60 (30% green light) on dry matter accumulated by lettuce leaves could be mainly attributed to the increase in leaf area caused by the shade avoidance syndrome, as described in this study. Kim et al. (2004) also found that fresh and dry weights of the lettuce shoot increased significantly when 24% of red and blue light was replaced with green light.

### Moderate Amount of Green Light Increased the Concentration of Carbohydrate and Nutritional Quality in Lettuce

Carbohydrate is the final product of CO_2_ absorption in the progress of plant photosynthesis (Taiz and Zeiger, 2015). In this experiment, the concentration of carbohydrates under the treatments employing green light was at a higher level than that of P_n−T_ in the difference of statistical analysis. This result implicated replacing red and blue light with 15–45% of green light since the ability of plants to accumulate carbohydrates was stronger under green light than under red and blue light.

In general, higher soluble sugar concentration results in a desirable taste of the plant (Lin et al., 2013). In this study, a relatively higher soluble sugar concentration was found under G60. Chen et al. (2016) also found that supplementing green light to white LEDs increased the soluble sugar content and decreased the nitrate content in green oak leaf lettuce. However, vegetables with high nitrate concentrations are harmful to human health. Especially in China, the allowable highest nitrate concentration of leaf vegetables is 3.0 mg g^−1^. In this study, the nitrate concentration of lettuce leaves could be significantly decreased by employing green light. Li et al. (2020) also found that partially replacing red and blue light with green light decreased the nitrate content in lettuce leaves. Bian et al. (2018) agreed with the above result that green light could decrease the nitrate contents in lettuce leaves by regulating the expression of some specific genes (e.g., *NR* and *NiR*). Crude fiber, as an important component of a plant cell wall, provided the crisp taste of and nutrients in lettuce for human beings (Taiz and Zeiger, 2015). However, no significant influence of green light on lettuce crude fiber was found in this study. Results presented in this study suggested that a moderate amount of green light could enhance the nutritional quality of lettuce plants.

## Conclusion

The optimal proportion of green light, partially replacing red and blue light, for promoting plant growth and development was investigated in this study. Our results showed that plant growth and morphology, light absorptance and transmittance, pigment concentration, stomatal characteristics, photosynthetic characteristics, carbohydrates, and nutritional quality were affected by different proportions of green light. The best green proportion was 30% in G60 in this study because G60 not only increased the leaf expansion and lettuce yield but also improved the accumulation of carbohydrate and nutritional quality. In addition, 15% green light in G30 and 45% green light in G90 also showed some advantages in lettuce growth. G30 increased the leaf expansion and yield and decreased the nitrate content of lettuce plants. G90 increased the accumulation of carbohydrates and decreased the nitrate content in lettuce plants, but no significant influence of G90 on lettuce yield was observed. Thus, from the perspective of economic benefits, the proportion of green light substituting red and blue light should be controlled between 15 and 30% in the production of lettuce, according to this experiment. The more accurate proportion still needs to be explored deeply in future studies.

## Data Availability Statement

The original contributions presented in the study are included in the article/Supplementary Material, further inquiries can be directed to the corresponding author/s.

## Author Contributions

LL carried out the measurements, data analysis, and drafted the manuscript. J-lL, Y-mL, and XL participated in part of measurements. Y-xT and R-fC made substantial guide about the experiment design and manuscript revision. All authors contributed to the article and approved the submitted version.

## Conflict of Interest

The authors declare that the research was conducted in the absence of any commercial or financial relationships that could be construed as a potential conflict of interest.

## Supplementary Material

Supplementary Figure 1 The diagram of the measuring apparatus.
