# Supplementary figures and images for: Morphology, Photosynthetic Traits, and Nutritional Quality of Lettuce Plants as Affected by Green Light Substituting Proportion of Blue and Red Light
Source: Front Plant Sci. 2021 Jul 7;12:627311. doi: 10.3389/fpls.2021.627311 (PMC8294060; doi:10.3389/fpls.2021.627311)

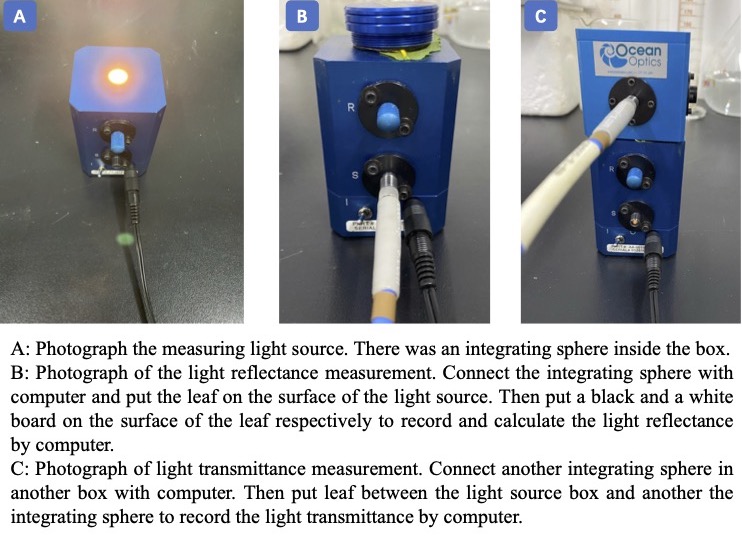

Supplement: Supplementary Figure 1 — The diagram of the measuring apparatus. [file Image_1.JPEG]
